# Determination of Agrin and Related Proteins Levels as a Function of Age in Human Hearts

**DOI:** 10.3389/fcvm.2022.813904

**Published:** 2022-03-09

**Authors:** Katie L. Skeffington, Ffion P. Jones, M. Saadeh Suleiman, Massimo Caputo, Andrea Brancaccio, Maria Giulia Bigotti

**Affiliations:** ^1^Bristol Heart Institute, Research Floor Level 7, Bristol Royal Infirmary, Bristol, United Kingdom; ^2^Institute of Chemical Sciences and Technologies “Giulio Natta” (SCITEC)-CNR, Rome, Italy; ^3^School of Biochemistry, University of Bristol, Bristol, United Kingdom

**Keywords:** agrin, proliferation, dystroglycan, extracellular matrix, myocardial infarction

## Abstract

**Background:**

Mature cardiomyocytes are unable to proliferate, preventing the injured adult heart from repairing itself. Studies in rodents have suggested that the extracellular matrix protein agrin promotes cardiomyocyte proliferation in the developing heart and that agrin expression is downregulated shortly after birth, resulting in the cessation of proliferation. Agrin based therapies have proven successful at inducing repair in animal models of cardiac injury, however whether similar pathways exist in the human heart is unknown.

**Methods:**

Right ventricular (RV) biopsies were collected from 40 patients undergoing surgery for congenital heart disease and the expression of agrin and associated proteins was investigated.

**Results:**

Agrin transcripts were found in all samples and their levels were significantly negatively correlated to age (*p* = 0.026), as were laminin transcripts (*p* = 0.023), whereas no such correlation was found for the other proteins analyzed. No significant correlations for any of the proteins were found when grouping patients by their gender or pathology. Immunohistochemistry and western blots to detect and localize agrin and the other proteins under analysis in RV tissue, confirmed their presence in patients of all ages.

**Conclusions:**

We show that agrin is progressively downregulated with age in human RV tissue but not as dramatically as has been demonstrated in mice; highlighting both similarities and differences to findings in rodents. Our results lay the groundwork for future studies exploring the potential of agrin-based therapies in the repair of damaged human hearts.

## Introduction

One major challenge in cardiovascular medicine is the loss of proliferative capacity of mammalian cardiomyocytes shortly after birth, rendering the adult heart unable to repair itself following an injury such as a myocardial infarction (1, 2). Much interest has therefore been focused on gaining a better understanding of the factors which prevent adult cardiomyocytes from proliferating, and whether it may be possible to positively affect cardiac repair by inducing their re-entry into the cell cycle. In an elegant series of experiments, Bassat and colleagues demonstrated that the cardiac extracellular matrix (ECM) plays an important role in controlling cardiomyocyte proliferation and identified the ECM proteoglycan agrin as a critical component of this process (3). Using a mouse model they found that the expression of agrin, a protein known to play a vital role in cardiac development (4, 5), is highest in the first days of postnatal life and significantly downregulated by day 7, which would exactly correspond to the timeframe over which murine cardiomyocytes lose the ability to proliferate (3, 6). Excitingly, administration of agrin to both murine and piglet models of myocardial infarction have been demonstrated to reactivate cardiomyocyte proliferation and promote cardiac repair (3, 7).

Agrin is primarily known for its fundamental role in recruiting/clustering of acetylcholine receptors at the neuromuscular junction (8, 9) and also for its genetic variants causing several forms of congenital myasthenia (10). However it has also been found to be involved in cancer pathogenesis, where it is thought to form a mechanotransductive link between the ECM and transcription factors YAP (Yes associated protein) and TAZ (transcriptional co-activator with PDZ binding motif), thus contributing to uncontrolled cell proliferation [reviewed in (11)]. The transcription factor YAP also promotes growth in cardiac tissue (12), and the work of Bassat and coworkers (3) suggests that, in neonatal cardiomyocytes, agrin plays a vital role in promoting cardiomyocyte proliferation through binding to the ECM protein dystroglycan (DG). DG is part of a larger transmembrane complex, the dystrophin-glycoprotein complex, and is composed by two subunits, the highly glycosylated extracellular α-DG and the transmembrane β-DG, that interact non-covalently to form a bridge between the ECM and the actin cytoskeleton (13, 14). It was hypothesized (3) that binding of agrin to α-DG in the myocardium causes a conformational change in the dystrophin-glycoprotein complex, which in turn prevents β-DG from binding YAP intracellularly, thus allowing its localization to the nucleus, where YAP can then exert its co-transcriptional activity and promote proliferation [reviewed in (15)]. Conversely, it has been demonstrated that in mature murine cardiomyocytes, YAP establishes a strong interaction with β-DG and is thus sequestered to the plasma membrane: the lack of nuclear localization impairs YAP co-transcriptional activity, resulting in loss of cardiomyocyte proliferation capacity (12). The mechanisms which cause YAP to become membrane bound and thus prevent proliferation are uncertain, but agrin expression has been observed to decrease significantly in the first days of postnatal life in murine species (3), in a timeframe that coincides with the loss of cardiomyocyte replicative capacity. It has been suggested that this may result in agrin being out-competed for binding to the DG receptor by another, more abundant protein such as the extracellular protein laminin [reviewed in (15)]. Replacement of agrin with a different interactor would trigger, in ways yet unknown, β-DG binding to YAP and its tethering at the cell periphery.

Whilst there are still many questions regarding the exact mechanisms of action involved, the possibility to harness the pro-proliferative capacity of agrin for regenerative purposes shows great potential. Crucially, all the work described has been performed in animal models, and currently nothing is known on whether similar pathways exist in human cardiac tissue, and how they are modulated during growth. A thorough analysis of the levels of agrin and of the proteins implicated in this proliferative axis at different ages in the human heart is the first necessary step to understand the potential of agrin for therapies to aid cardiac regeneration in humans. Such an analysis is hindered by the intrinsic difficulties connected to the collection of healthy human cardiac samples. In this study we have used right ventricular biopsies collected from patients undergoing cardiac surgery to investigate for the first time how the levels of agrin, DG, laminin and YAP change with age in human heart tissue.

## Materials and Methods

### Sample Collection

Waste right ventricular tissue was collected from 40 patients undergoing cardiac surgery at the Bristol Royal infirmary for a variety of congenital cardiac conditions (Table 1). Full informed consent was obtained prior to surgery from the patients or their parents as appropriate. The study was conducted in accordance with the declaration of Helsinki, and the protocol was approved by the North Somerset and South Bristol Research Ethics Committee (REC 07/H0106/172). Half of the collected tissue from each patient was fixed in 10% formalin before being transferred to PBS for storage at 4°C. The other half of the tissue was stored at −20°C in Allprotect Tissue Reagent (Qiagen). Heart tissue lysates for 0 days old and adult mice were purchased from Leinco Technologies (cat.# M1135 and M1013, respectively). Formalin-fixed mid-cardiac section from 3 month old adult mice were obtained from Insight Biotechnologies (cat.# MoFPT016).

**Table 1:** Patient characteristics.

**Pathology**	**Number of patients**	**Age in years (mean ±SEM, median, max, min)**	**Gender (M/F)**
TOF	15	1.03 ± 0.22, 0.59, 2.79, 0.23	8/7
VSD	8	0.27 ± 0.05, 0.32, 0.38, 0.02	4/4
PVR	9	12.16 ± 1.97, 12.66, 24.29, 4.25	8/1
Other	8	7.85 ± 5.50, 0.11, 43.93, 0.003	4/4
**All**	**40**	**4.75** **±1.37, 0.57, 43.93, 0.003**	**24/16**

*Patients characterized by prevalent diagnosis/surgery*.

*TOF, Tetralogy of Fallot; VSD, ventricular septal defect; PVR, pulmonary valve replacement*.

*“Other” includes patients undergoing Ross-Konno procedures and patients with cardiac tumors, Truncus Arteriosus or Total Anomalous Pulmonary Venous Drainage*.

### mRNA Analysis

Total RNA was extracted from tissue samples stored as described above using the RNeasy Mini Kit. Specifically, 5–15 mgs of RV tissue from each patient were homogenized according to the manufacturer instructions using PowerBead Tubes filled with 0.7 mm garnets (Qiagen) to efficiently break the tissue by 4–6 × 1 min cycles in a mechanic vertical shaking homogenator (Minilys, Bertin Technologies) at 4°C. The integrity of the RNA thus prepared was confirmed by the Agilent Tape station RNA assays on an Agilent 2,200 TapeStation instrument, and RNA Integrity Numbers (RIN) were calculated by the machine software. The quantity of extracted RNA was determined by measuring the absorbance at 260 nm on a nanodrop (Thermo Scientific), and RNA quality was deemed good for downstream analysis when Abs260 nm/Abs280 ≥ 1.8 and Abs260 nm/Abs230 ≥ 2. 0.5–1 μg of total RNA from each patient were reverse transcribed using the QuantiTect Reverse Transcription Kit (Qiagen). When necessary, the RNA was concentrated before reverse transcription (GeneJET RNA Cleanup and Concentration Micro Kit, Thermo Scientific).

RT-PCR reactions were run on a QuantStudio5 Real Time PCR machine (ThermoFisher Scientific), using the QuantiFast SYBR Green PCR Kit (Qiagen). Target and housekeeping RNAs were amplified with the following QuantiTect Primer Assay primer mixes (Qiagen): Hs_AGRN_va.1_SG (agrin), Hs_DAG1_1_SG (DG), Hs_LAMA2_1_SG (laminin-α2), Hs_YAP1_1_SG (YAP), Hs_GAPDH_1_SG (GAPDH), HS_IPO8_1_SG (IPO8) and HS_POLR2A_1_SG. The α2 chain was chosen as representative of laminin in the heart, where it is one of the most populated forms (16). Five nanograms of cDNA from the retrotranscribed material were used in each reaction, and all reactions were performed in triplicate in each experiment. The data reported in the results were averaged over a minimum of 2 RT-PCR reactions for each patient. To take into account the heterogeneity of the RV samples, based on the work of Molina and colleagues (17) three different genes stably expressed in human cardiac tissue were chosen as reference for determining all the ΔCt values, namely glyceraldehyde 3-phosphate dehydrogenase (*GAPDH*), importin 8 (*IPO8*) and RNA polymerase II subunit A (*POLR2A*). Comparative quantification was used to identify whether the target genes were up- or down-regulated relative to the two oldest patients under analysis. Relative expression of all genes was calculated by the ΔΔCt method. Specifically, if Ct(TP) is the Ct value measured for a target gene in a patient (TP), and Ct(AvgeRP) is the averaged Ct of the 3 reference genes for that patient, then the relative expression for the patient (ΔCtTP) is calculated as:


$$\begin{array}{l} \text{Δ}CtTP\;=\;Ct\;(TP)\;-\;Ct(AvgeRP) \end{array}$$

Up- or down-regulation of target genes were assessed relative to the average expression in the two oldest patients (P'), aged 43 and 24, so that:


$$\begin{array}{l} \text{ΔΔ}CtTP=\text{Δ}CtTP-\text{Δ}CtTP^{\prime } \end{array}$$

Throughout the paper, transcripts levels are expressed as fold changes relative to the average expression of the two oldest patients, as calculated from the expression:


$$\begin{array}{l} Fold\;change=2^{-\text{ΔΔ}CtTP} \end{array}$$

### Immunohistochemistry (IHC)

The fixed tissue was embedded in paraffin and sectioned at 4 μm thickness. The sections on the slides were deparaffinized and antigen retrieval performed using 10 mM citrate buffer, pH 6.4, at 95°C. The slides were washed in PBS, endogenous peroxidase activity inhibited by a 10 min incubation with Bloxall (Vector Laboratories), and the slides rewashed. The sections were then incubated for 30 min at room temperature in 10% donkey serum (Sigma Aldrich) to block non-specific antigens, followed by incubation in primary antibody (agrin: GTX54904, GeneTex, laminin-α2: STJ93889, St John's Laboratory, DG: 11017-1-AP, Proteintech) or PBS (negative control) overnight at 4°C. The sections were then washed in PBS and exposed to secondary antibody (ECL anti-rabbit IgG, NA934, GE Healthcare) for 1 h at room temperature before another wash and exposure to DAB (Vector Laboratories) for 20 min. Finally, the slides were counterstained with Mayer's Haemotoxylin and images were taken using an O8 slidescanner (Precipoint).

### Proteins Identification

Total proteins were extracted from samples stored at −20°C in Allprotect Tissue Reagent (Qiagen). RIPA lysis buffer with freshly added phosStop (Roche 04906837001), cOmplete protease inhibitors cocktail (Roche 11836170001) and benzonase (Thermo Scientific) was added to 10–20 mg of samples into gentleMACS M Tubes with strainers (Miltenyi Biotech). The tissue was then homogenized into a gentleMACS Dissociator instrument (Miltenyi Biotech). Insoluble material in the homogenates was spun down by centrifugation (5 min at 4,000 xg, 4°C), the supernatants were checked for total protein content (see below) and stored at −80°C. The extracted proteins in each sample were quantified with the Pierce™ Rapid Gold BCA Protein Assay Kit (Thermo scientific: A53225) following the manufacturer instructions.

#### Blotting

Total proteins for each sample were diluted to load an amount of 20 μg/gel lane and denatured by adding LDS loading buffer (Invitrogen). Samples were loaded on 4–12% Bis-Tris Gels (Invitrogen) and SDS-PAGE were run at 100V in MES SDS running buffer. The Spectra Multicolour Broad Range Protein ladder was used as a molecular weight marker (Thermo Scientific). Proteins were transferred on activated PVDF membranes (Thermo scientific) at 250 mA for 1.30 h at room temperature (RT). After blocking and washing, blots were hybridized with primary antibodies overnight at 4°C; see Supplementary Table 1 for details on the antibodies used. To note, due to inefficient probing with the anti-laminin-α2 Ab, an anti-laminin-γ1 Ab was used instead (see Supplementary Table 1). Blots were then washed and incubated 1 h with the secondary antibodies indicated in Supplementary Table 1. Blots were developed using 1 ml of Immobilon Forte Western HRP substrate (Millipore- WBLUF0500) for 1 min. For each membrane both colorimetric and chemiluminescence images were taken on a ChemiDoc MP imaging system (BioRad) and analyzed with the BioRad Image Lab software. When necessary, membranes were stripped using Restore^TM^ Plus Western blot stripping buffer (Thermo scientific) according to the protocol provided and re-probed with the antibodies of choice. Densitometric analysis, when possible, was conducted using ImageJ.

#### Liquid Chromatography Mass Spectrometry

A total of 50 ug of total protein extract from each RV tissue lysate were subjected to tryptic digestion followed by a LC-MS run on an Orbitrap Fusion Tribrid Mass Spectrometer with ETD instrument (Thermo Scientific) operated with up-stream Ultimate 3,000 nano-LC system at the Proteomic Facility at the University of Bristol. The resulting data were analyzed with a Sequest search against the Uniprot Human database and against an in house database of common contaminants, and the results have been filtered using a 5% FDR cut-off.

### Statistics

Correlations between RNA expression and age were assessed using two-tailed Pearson correlation coefficient. Gender differences were assessed using unpaired Student's *t*-tests and differences between pathologies using one-way ANOVA. Regression analysis was used to find the unstandardised regression coefficient β and it's standard error for agrin expression in each pathology group. All statistics were performed in SPSS Version 26 (IBM Analytics, New York, NY) and significance was accepted when *p* < 0.05.

## Results

**Figure 1:**
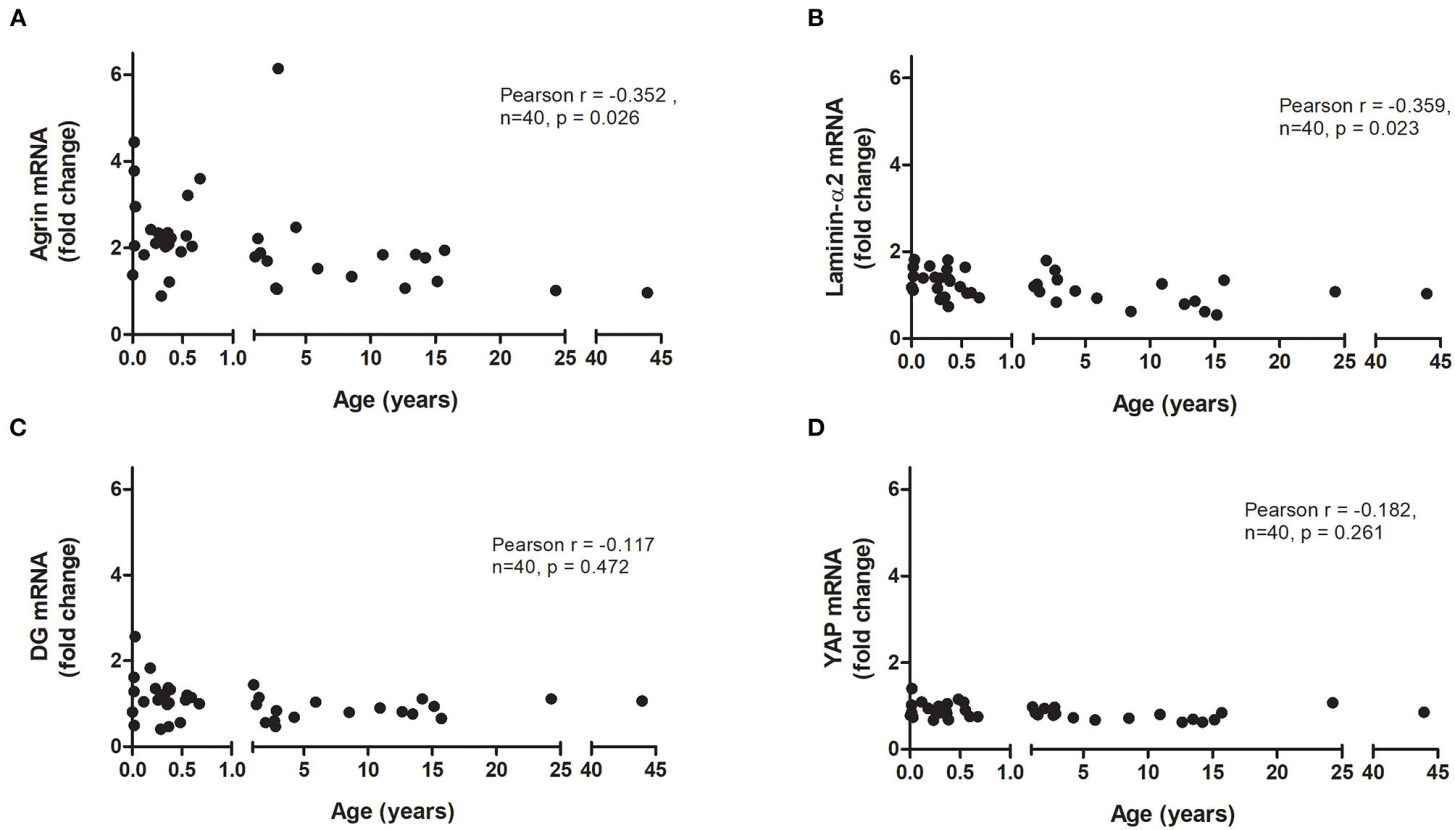
Relative mRNA expression of agrin and associated proteins in human hearts. Correlation of the transcript levels of agrin **(A)**, laminin **(B)**, dystroglycan (DG; **C**) and YAP **(D)** with age in right ventricular biopsies from patients undergoing cardiac surgery (*n* = 40). Fold changes were calculated relative to the mRNA levels of two adult patients (24 and 43 years). Significant correlations were tested for using two-tailed Pearson correlation coefficient.

Forty patients [mean age 4.7 ± 1.4 years (mean ± SEM)] undergoing cardiac surgery for a variety of conditions were recruited (Table 1). The RNA obtained from the tissue was of high quality, with RIN values in the range of 8–9. The expression of agrin, laminin-α2, DG and YAP were very similar between the 24 year old and 43 year old (Figure 1), and the results of the other patients were normalized to the average expression in these two adult patients. The agrin gene was found to be expressed in the right ventricle of all patients, with its transcript levels (expressed both as ΔΔCts relative to the adult controls and as individual ΔCts) significantly negatively correlated to the patient age at the time of operation (*p* = 0.026, Figure 1A and Supplementary Figure 1B, respectively). As an immediate measure, upon correction/normalization against the housekeeping controls, the number of RT-PCR cycles of neonates (highest expression, i.e., lowest RT-PCR cycles) is 1.55 times lower than those of the two adults (lowest expression, i.e., highest RT-PCR cycles).

Splitting the pediatric cases into two age groups (0–1 years and 1–15 years) demonstrates that it is not only babies who have higher agrin expression than adults; agrin levels also remain elevated in older children (Supplementary Figure 1A). The α2 chain was chosen as representative of laminin in the heart, where it is one of the most populated forms (16), and its expression also demonstrated a significant negative correlation with patient age (*p* = 0.023, Figure 1B and Supplementary Figure 1C). The expression of DG and YAP, on the other hand, did not correlate with age (Figures 1C,D and Supplementary Figures 1D,E).

**Figure 2:**
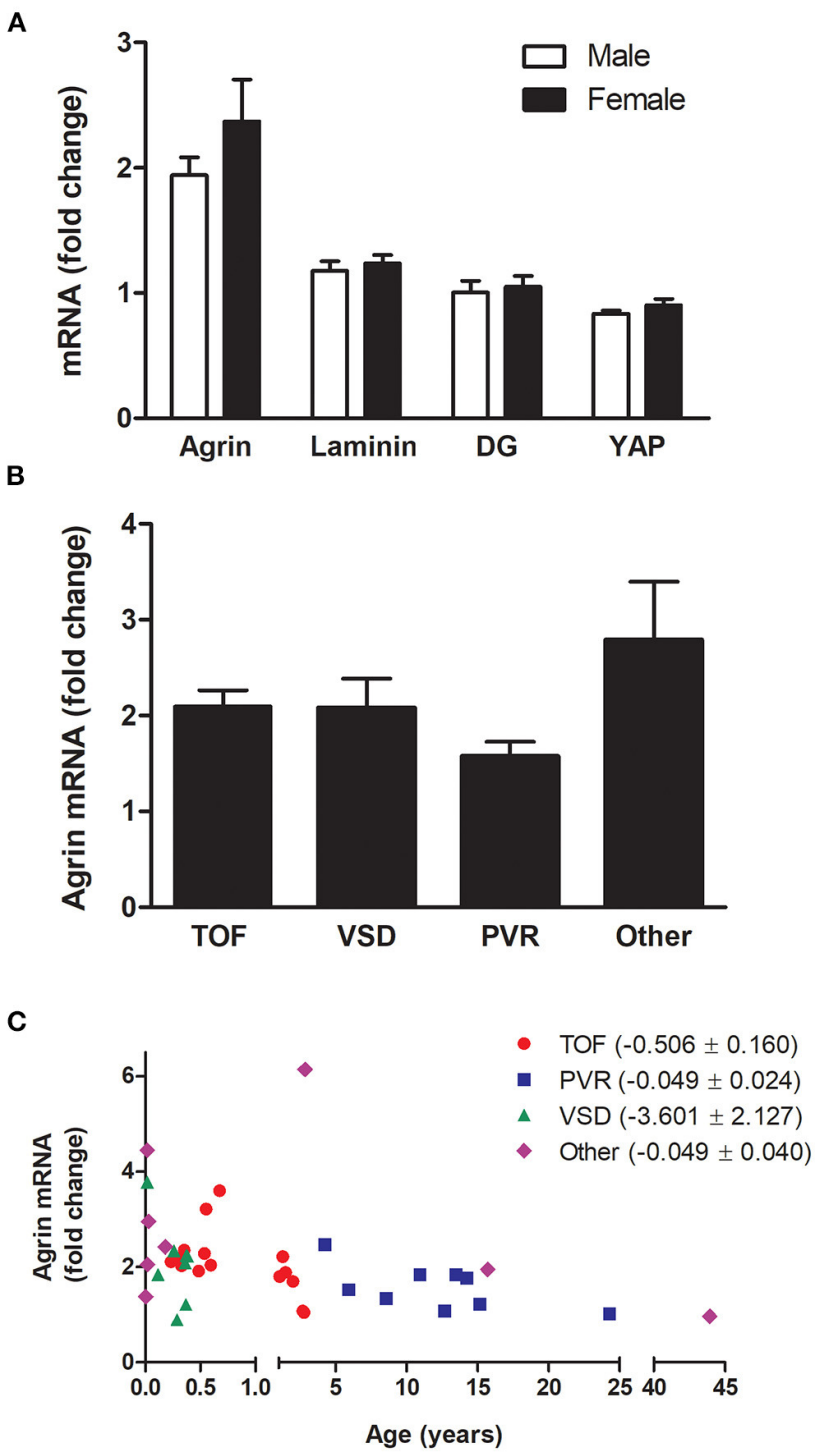
mRNA expression of agrin and associated proteins by gender and pathology. Transcript levels for agrin, laminin, DG and YAP in male patients compared to females **(A)** and transcript levels of agrin in patients with different pathologies **(B,C)** *n* = 40. In particular, **(C)** shows the agrin levels dependence on age as broken down according to pathology. TOF, Tetralogy of Fallot; VSD, ventricular septal defect; PVR, pulmonary valve replacement. “Other” includes patients undergoing Ross-Konno procedures and patients with cardiac tumors, Truncus Arteriosus or Total Anomalous Pulmonary Venous Drainage. The bracketed numbers in the legend are the unstandardised coefficient β ± SEM. Fold changes were calculated relative to the mRNA levels of two adult patients (24 and 43 years old). Student's *t*-tests for unpaired data **(A)** or one-way ANOVA **(B)** revealed no significant differences.

The relative transcript levels of agrin, laminin, DG or YAP were not significantly different between male and female patients (Figure 2A), nor any significant difference was found between groups of patients with different pathologies (Figure 2B). In this respect, it has to be noted that the distribution of ages in the 4 main categories of pathology, although partially overlapping, is different, with patients with ventricular septal defects being the youngest, followed by Tetralogy of Fallot patients and with the eldest patients grouped in the pulmonary valve replacement and “other” categories (see Table 1). Remarkably, the levels of agrin decrease with age in each of the categories (Figure 2C), thus strongly indicating that the phenomenon is independent from pathology in the cohort of patients under consideration. Furthermore, analysis of the other clinical data available for the whole cohort of patients highlighted the absence of significant correlation between agrin levels and any of the collected clinical parameters.

**Figure 3:**
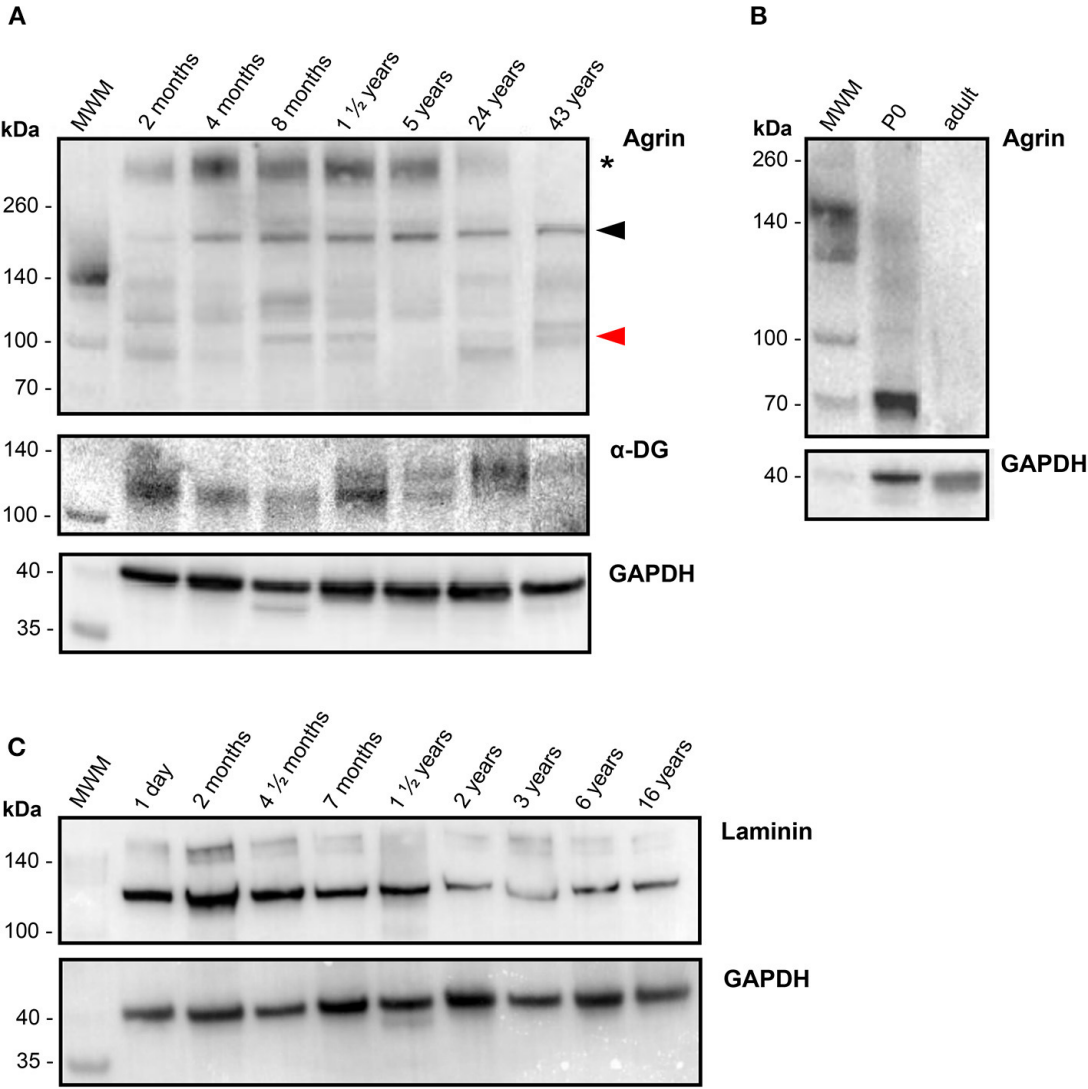
Protein levels of agrin and related proteins in human hearts. **(A)** Western blots of the proteins indicated, as detected in RV samples collected from representative patients (*n* = 7) of different ages, in increasing order from left (2 months old) to right (43 years old) as identified on top of each lane. For comparison, a western blot of agrin detected in commercial mouse heart tissue lysates (Leinco Technologies) from 0 days old (P0) and adult mice is reported in **(B)**, together with the corresponding loading control GAPDH at the bottom. **(C)** Western blots of laminin (plus loading control), as detected in RV samples collected from representative patients (*n* = 9) of different ages, in increasing order from left (1 day old) to right (16 years old) as identified on top of each lane Proteins extracted from human RV samples as well as the commercial mice heart samples were probed with the antibodies specified in Supplementary Table 1, and bands of the correct molecular weight have been identified for laminin-γ1 (~160 kDa), α-DG (110-130 kDa) and the housekeeping protein GAPDH (36 kDa). The series of bands identified for human agrin originate from differential glycosylation and proteolytic degradation: the asterisk indicate the fully glycosylated form (~400 kDa), the black arrowhead points to a band that could represent the unglycosylated form (~220 kDa) and the red arrowhead indicates the ~110 kDa band that could represent a C-terminal agrin fragment (CAF), see Results for details.

To investigate the presence and possible age-dependence of agrin, laminin and DG at the protein level, total protein extracts from the 40 human RV samples were analyzed by western blotting. Figure 3 reports western blots of representative samples set out in increasing age order from left to right for the three proteins (Figures 3A,C). The blots support the qPCR results, showing that agrin is expressed in human heart throughout life, as shown by the presence of clearly defined bands from the youngest representative (2 months) RV sample all the way through to the oldest (43 years). For comparison, Figure 3B shows a western blot on heart extracts from mice, whereby agrin is present in neonatal heart but undetectable in adult. Laminin and dystroglycan are also abundant in all the samples, with no significant variability in the amount of both proteins across different age groups. Although a proper quantification was hindered by a considerable heterogeneity in the agrin bands (due to extensive glycosylation and possibly to a certain amount of degradation upon samples preparation) the protein was consistently detected in replicate WB experiments in all the patients. Moreover, a high molecular weight band, representing the fully glycosylated form of agrin (~400 kDa, see black asterisk in Figure 3A) was detected in samples of all ages and, although not properly quantifiable by densitometry, a trend of decreasing levels from youngest to oldest patients is evident, Notably, agrin has been found to be intrinsically prone to proteolysis in skeletal muscle atrophy/wasting induced by stroke (18) and chronic heart failure (19) and the 110 kDa C-terminal agrin fragment thus produced constitutes a biomarker of muscle wasting. Interestingly, one of the populated bands in our agrin western blots has an apparent molecular weight of ~110 kDa (Figure 3), compatible with that of the C-terminal agrin fragment. Although no obvious effect of age on the levels of the 110 kDa fragment is evident, other factors could affect this and other fragment populations, including any prior heart injuries and the variable degree of degradation of the samples, thus making any effect of age less detectable. Since sample extracts from the 40 RV samples analyzed in this study presented different degrees of degradation, densitometric analysis based on all of the detectable bands could not be conclusive, and no direct comparison could be drawn with transcriptomic results. Also interestingly, inspection of the α-DG bands detected on the blot reveals an opposite trend of glycosylated protein as compared to agrin, i.e., α-DG appears to be more glycosylated with age. Lastly, since the laminin signal in the blots shown in Figure 3C appeared the most reliable in terms of opportunity to quantify it, a densitometric analysis was conducted and confirmed that laminin levels (at least those of laminin-γ1, as probed on the western blots) decrease with age of a factor of 3.5 folds (see also Supplementary Figure 2), which is in line, although slightly higher, with the transcripts evidences.

LC-MS has also been employed in an attempt to identify and make a semi-quantitative assessment of the proteins under analysis. Due to the scarcity of the starting material for all the samples, no pre-fractionation of the protein extracts was possible, hindering a precise quantification. Nonetheless, protein lysates of RV tissue from 2 representative patients (2 months and 24 years old) were subjected to a LC-MS analysis upon proteolytic digestion. The results, summarized in Supplementary Table 2, show how agrin, laminin and α-DG were all identified with a high degree of confidence in both samples, despite ECM proteins being notoriously difficult to characterize with this kind of technique. Interestingly, although the hits for α-DG are comparable in the two samples analyzed, the number of peptides identified for agrin and laminin (and thus the total sequence coverage for both) are much higher in the RV sample from the infant than that from the adult patient. Specifically, although no definitive conclusions can be drawn for laminin, the sensibly smaller representation of agrin peptides in the older patient might indicate a smaller amount of full-length protein, possibly due to a lower level of expression as well as a higher degree of degradation with respect to the younger patient.

**Figure 4:**
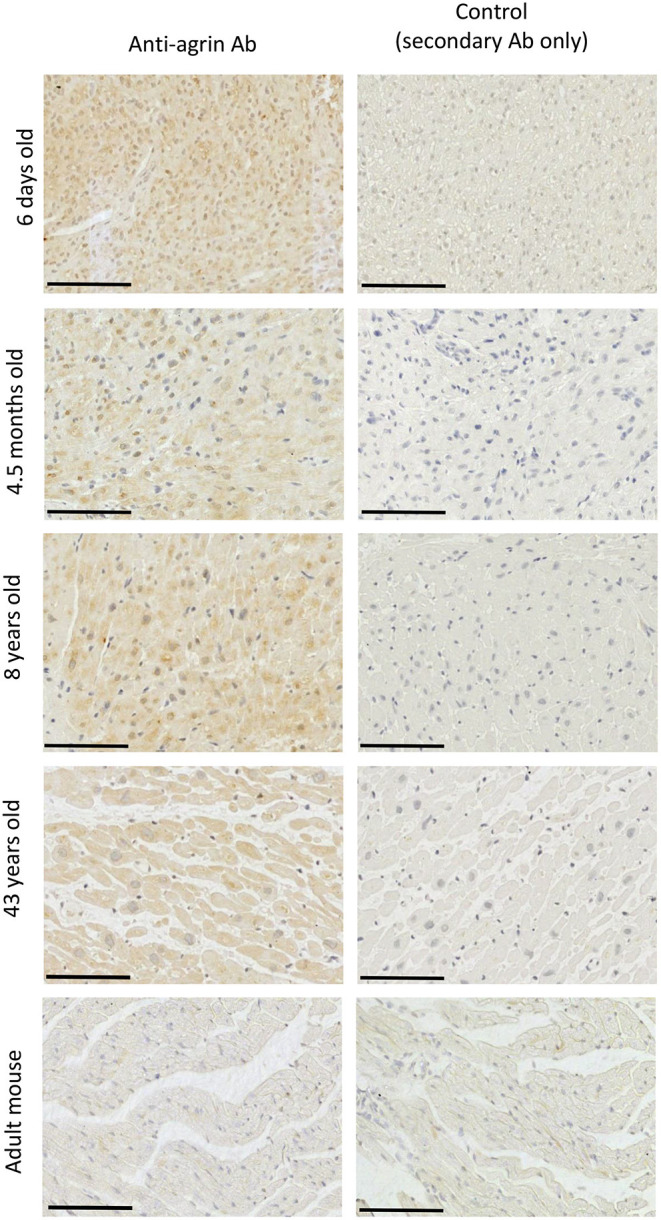
Identification of agrin in RV tissue sections. Immunohistochemical staining for agrin in the RV of four patients aged between 6 days and 43 years and in the RV of an adult mouse (9 weeks). Each scale bar represents 100 μm. A negative control for each image is found in the right-hand column.

IHC staining aimed at identifying on-tissue the proteins under analysis, found evidence of the expression of agrin within the ECM in tissue from patients of all ages (Figure 4). The protein appears to have a wide distribution, with staining of cardiomyocytes particularly evident in adult human samples. For comparison, we performed similar IHC experiments in adult mice hearts (Figure 4, bottom panels), where we found very little evidence of agrin staining. Supplementary Figures 3, 4 show a similar distribution of laminin and DG mainly within the ECM of cardiomyocytes in samples of the whole age range. It is important to stress that, like western blots in the specific conditions here described, IHC can only be used as a qualitative indicator of the presence and localization of the three proteins in the tissues under analysis and as such could not provide information to complement (or otherwise) the quantitative transcriptomic results. Nevertheless, the results of transcripts and proteins analysis are consistent in showing how agrin is the least populated of the proteins under investigation, as indicated by the higher ΔCt values (indicative of lower mRNA levels, see Supplementary Figure 1) and the relatively fainter signals in western blots (see Figure 3) as well as IHC (Figure 4 vs. Supplementary Figures 3, 4, respectively) for agrin relative to both DG and laminin. Finally, although little is known about agrin presence in the heart in locations other than the ECM of the cardiac muscle, it is likely that, by analogy to skeletal muscle, there may also be agrin at neuromuscular junctions. Since the cardiac muscle, which is the source of the samples used in this work, is mainly composed of cardiomyocytes [70% of myocardium volume (20)], it is reasonable to assume that the agrin analyzed in this work comes from the ECM of cardiomyocytes and from neuromuscular junctions.

## Discussion

Our study is the first to collect data on the transcription/expression levels of agrin and associated proteins in human cardiac tissue at different stages of development. Previous work from a different laboratory demonstrates that, in mice, agrin transcript levels halve in the first week of postnatal life and continue to decrease into adulthood (3); an even stronger negative time dependency was shown for the protein product, with agrin protein expression in adult mice around one tenth of that found in neonates. However, a study by a different group using only immunofluorescence staining reported similar levels of agrin expression in adult and embryonic mouse hearts (21). Our own results in mice suggest a decrease in expression of the agrin protein with age and very little agrin presence at the protein level in adult mice as measured both by IHC and western blotting. Our transcripts results on human cardiac tissue demonstrate that there is also a decrease in agrin expression with age in human hearts. This finding lays the groundwork for a molecular analysis aimed at understanding whether this change in agrin expression with age is associated with a role for this protein in cardiac regeneration, similarly to what has been described in mice. Such an important analogy, however, has its counterpart in the evidence that agrin expression, crucially, does not decrease with age in humans as dramatically as it has been described in mice. Indeed we measured an agrin transcripts fold change in neonates that is 2.3x the levels found in adults, suggesting a reduction of agrin expression of only slightly more than half with growth to adulthood in humans. Furthermore, the reduction in agrin expression with age is even less apparent at the protein level, with agrin being clearly present in tissue sections of adult controls, where staining for agrin in IHC is comparable to that recorded on samples from much younger patients (see Figure 4); this is in striking contrast with the almost complete lack of agrin signal we measured in RV sections from adult mice. The results of the western blot go in the same direction, with comparable levels of protein measured in samples of all ages, irrespective of the degradation pattern, which appears to be rather sporadic and non-specific within the wide age range considered. On the other hand in mice, similarly to the IHC data, there is almost no agrin detectable by western blot at adulthood (Figure 3B). Thus our data demonstrate a less dramatic reduction in agrin expression with age than is seen in mice, both at the transcript level and even more so at the protein level. If a role for agrin in a possible regenerative program was confirmed in humans, these results may point to important differences in how it is controlled in rodent vs. human hearts.

It has been suggested that, with age, the binding of agrin to the α-DG receptor may be increasingly outcompeted by another extracellular protein, and laminin has been put forward as a potential candidate for this (15). Laminin is known to play an important role in promoting cardiac muscle stability (22) as well as being crucial for cardiomyocyte differentiation (23, 24) and maturation (25). Laminin and agrin show a similar affinity for α-DG (26), and a slight increase in laminin expression has been demonstrated between postnatal days 1 and 2 in mice (6). However, results in the current study suggest that in human cardiac tissue (similarly to what was found with agrin) the expression of laminin in the right ventricle is negatively correlated with age. Interestingly, using an analogous approach to the one we have employed (but relying on post-mortem myocardial biopsies), it was shown that, irrespective of the presence of a cardiac pathology, there was a negative correlation between age and laminin-α2 transcripts in a cohort of human subjects between 9 and 58 years old (27). The correlation we found of laminin expression with age was indeed more significant than the correlation of agrin with age (*p* = 0.023 and *p* = 0.026, respectively), however this comparison is affected by the presence of one patient who had unusually high agrin expression for their age (Figure 1A), weakening the strength of the correlation. As a matter of fact, the decrease in laminin-α2 transcript levels with age in our study was fairly modest, and it is unclear how much functional significance it would have. On the other hand, quantification at the protein level shows a decrease of about 3.5 folds in the adults relative to infants, which needs to be further investigated. Although purely observational in nature, our findings would not seem to confirm the hypothesis formulated by Bassat and coworkers (3), that a simultaneous reduction in agrin expression and increase in laminin levels with age results in the latter out-competing agrin in binding to α-DG. However, we show that the negative correlation of agrin expression with age is steeper than that of laminin in human RV (Figures 1A,B), and since the measured α-DG binding affinities of the two proteins are very similar (26), this could account for a possible replacement of agrin with laminin as early in life as by the first year of age. Notably, our results also show for the first time that, although α-DG expression in RV tissue is not significantly correlated with age, its level of glycosylation might be, as demonstrated by the higher apparent molecular weight of the glycosylated species of adults as compared to children in our western blot analysis. Taken together with the finding that the trend in glycosylated agrin with age is the opposite, i.e., adults show a less glycosylated protein than children, this observation could suggest a possible role of the reciprocal glycosylation states of α-DG and agrin in their interaction along the growth of individuals.

Our investigation of the protein levels by IHC and western blots, although less quantitative than our transcriptomic analysis, confirms that agrin is present in the cardiac tissues of all the patients analyzed, therefore also suggesting that the agrin/laminin expression levels during cardiac muscle development and in adulthood are not controlled in the “all or nothing” way described in mice. Moreover, our western blot analysis confirms the relatively high susceptibility of the C-terminal portion of agrin to proteolysis, as has been previously shown in skeletal muscle (18, 19). It could be hypothesized that such degradation, by negatively affecting the interaction of agrin with DG, is a factor in the loss of pro-proliferative capacity of this protein along the lifetime of an individual, although such an hypothesis is possibly too simplistic, based on the evidence of the efficacy of recombinant agrin (composed exclusively of the ~100 kDa C-terminal) in regenerating the heart of animal models (3, 7).

It is possible that additional factors are involved which favor the binding of the α-DG receptor to laminin over agrin. For example, in skeletal muscle, changes in the degree of glycosylation of the α-DG receptor can affect the affinity of laminin for the receptor, resulting in muscular dystrophy (28, 29). Despite our preliminary evidences of a possible positive trend of glycosylation with age, it is fundamentally unknown whether the degree of glycosylation of the α-DG receptor in cardiac tissue changes with age, and to what extent this might affect the binding of agrin or laminin. Alternatively, laminin may not be the only other key player at all and other, as yet unidentified, proteins or factors that influence the binding affinity of agrin to the α-DG receptor may be involved. Interestingly, DG has been shown to also play a fundamental role in actively inhibiting the spread of myocardial damage from cardiomyocytes to neighboring cells (30), which points to the capacity of this receptor to sense the extracellular environment and respond to the cellular needs and might hint to its direct involvement in controlling the expression/action of agrin. Additionally, it has been demonstrated that cardiac maturation also results in the phosphorylation of YAP via its interaction with proteins in upstream pathways such as the Hippo pathway (31, 32), and the relative importance of YAP phosphorylation vs. the interaction of agrin with α-DG in leading to the sequestration of YAP at the cell periphery is unknown.

There has been much interest in the potential use of agrin based therapies in cardiac regeneration. Until now all work has however been carried out in animal models. This study demonstrates for the first time that agrin is actively expressed in human cardiac tissue in an age-dependent way, with a transcripts peak very early in life and a steady decrease toward adulthood, echoing the pattern of expression observed in rodents (3). Future work is necessary to identify a possible agrin-driven regenerative program, elucidate the molecular mechanisms involved and investigate whether these pathways could be harnessed therapeutically to promote cardiac regeneration in humans. In this respect, our finding that agrin is clearly detectable at the protein level in adult human heart shows how, unlike what described for rodent hearts, if an agrin-driven proliferative program exists in humans, it does not fully switch off with aging, opening up new therapeutic scenarios. Indeed, a potential pharmacological re-activation of the endogenous agrin-DG axis could constitute an alternative/complementary approach to the direct administration of exogenous agrin proven to be effective in cardiac repair of murine (3) and porcine (7) models.

One limitation of this study is that all the cardiac tissue analyzed came from patients with diseased hearts, since for obvious ethical reasons it is not possible to obtain fresh cardiac tissue (*ex-vivo*) from healthy human hearts. However, as discussed above the patients involved in this study suffered from a variety of cardiac conditions, and there was no evidence of an association of altered agrin expression with any particular pathology, nor with gender or any of the recorded clinical parameters. Another limitation is that all samples came from the right ventricles of patients. Although it cannot be ruled out that the scenario described here may be different in the left ventricle, studies that have investigated the differential characteristics of left and right ventricles at the molecular level have not highlighted any major differences between the two in the agrin related pool of proteins that are the object of this analysis (33).

## Data Availability Statement

The original contributions presented in the study are included in the article/Supplementary Material, further inquiries can be directed to the corresponding authors.

## Ethics Statement

The studies involving human participants were reviewed and approved by North Somerset and South Bristol Research Ethics Committee (REC 07/H0106/172). Written informed consent to participate in this study was provided by the participants' legal guardian/next of kin.

## Author Contributions

MB and AB conceived the project. MB, AB, MS, and MC conceptualized and designed the research. MC collected the clinical samples. KS, FJ, and MB performed the research. KS, FJ, AB, and MB analyzed the data. KS and MB drafted the manuscript, which was edited and approved by all authors. All authors contributed to the article and approved the submitted version.

## Funding

This work was supported by grants from the Sir Jules Thorn Charitable Trust (MC) and the British Heart Foundation, grant no. CH/1/32804 (KS, MB, and MC). FJ is the recipient of a British Heart Foundation PhD studentship in Integrative Cardiovascular Sciences. The Cardiovascular theme of NIHR Bristol Biomedical Research Centre also supported this work. The funders played no role in the design of the study, in the collection, analysis and interpretation of data, or in the decision to submit the manuscript for publication.

## Conflict of Interest

The authors declare that the research was conducted in the absence of any commercial or financial relationships that could be construed as a potential conflict of interest.

## Publisher's Note

All claims expressed in this article are solely those of the authors and do not necessarily represent those of their affiliated organizations, or those of the publisher, the editors and the reviewers. Any product that may be evaluated in this article, or claim that may be made by its manufacturer, is not guaranteed or endorsed by the publisher.

## Abbreviations

DG: Dystroglycan
ECM: Extracellular matrix
IHC: Immunohistochemistry
RV: Right ventricle
YAP: Yes associated protein.
