# Supplementary material for: Determination of Agrin and Related Proteins Levels as a Function of Age in Human Hearts
Source: Front Cardiovasc Med. 2022 Mar 9;9:813904. doi: 10.3389/fcvm.2022.813904 (PMC8959542; doi:10.3389/fcvm.2022.813904)
Supplement: Supplementary file 1 [file Data_Sheet_1.docx]

**Supplementary Table S1. Details of the antibodies used in Western blotting.**

| **Protein of interest** | **Primary antibody** | **Concentration** | **Secondary antibody** | **Concentration** |
| --- | --- | --- | --- | --- |
| Agrin | Gene Tex, Human Agrin antibody, antigen affinity-purified polyclonal Rabbit. Cat no. GTX54904 | 1:5000 | Pierce primary Goat Anti-Rabbit IgG; H+L peroxidase conjugated. Cat no. 31466 | 1:5000 |
| α-Dystroglycan | R&D Systems, Human (DG) antibody, antigen affinity-purified polyclonal sheep. Cat no. AF6969. | 1:200 | CUSABIO, Rabbit anti-Sheep IgG Antibody; HRP conjugated. Cat no. CSB-PA00110F1Rb. | 1:6000 |
| GAPDH | Gene Tex, Human GAPDH antibody, antigen affinity-purified polyclonal Rabbit Cat no. GTX100118. | 1:12000 | Pierce primary Goat Anti-Rabbit IgG, (H+L) conjugated. Cat no. 31466 | 1:12000 |
| Laminin-γ1 | St John’s Laboratory, Anti-laminin γ-1 antibody. Cat no. STJ93896) | 1:5000 | Pierce primary Goat Anti-Rabbit IgG, (H+L) conjugated. Cat no. 31466 | 1:5000 |

**Supplementary Table S2. Details of the LC-MS analysis.**

Colour code: Cyan = α-DG; Yellow = Agrin; Green = Laminin.

The data within the top 9 rows report on a 2 months old patient, the bottom 3 rows are relative to the adult patient (24 years)

**
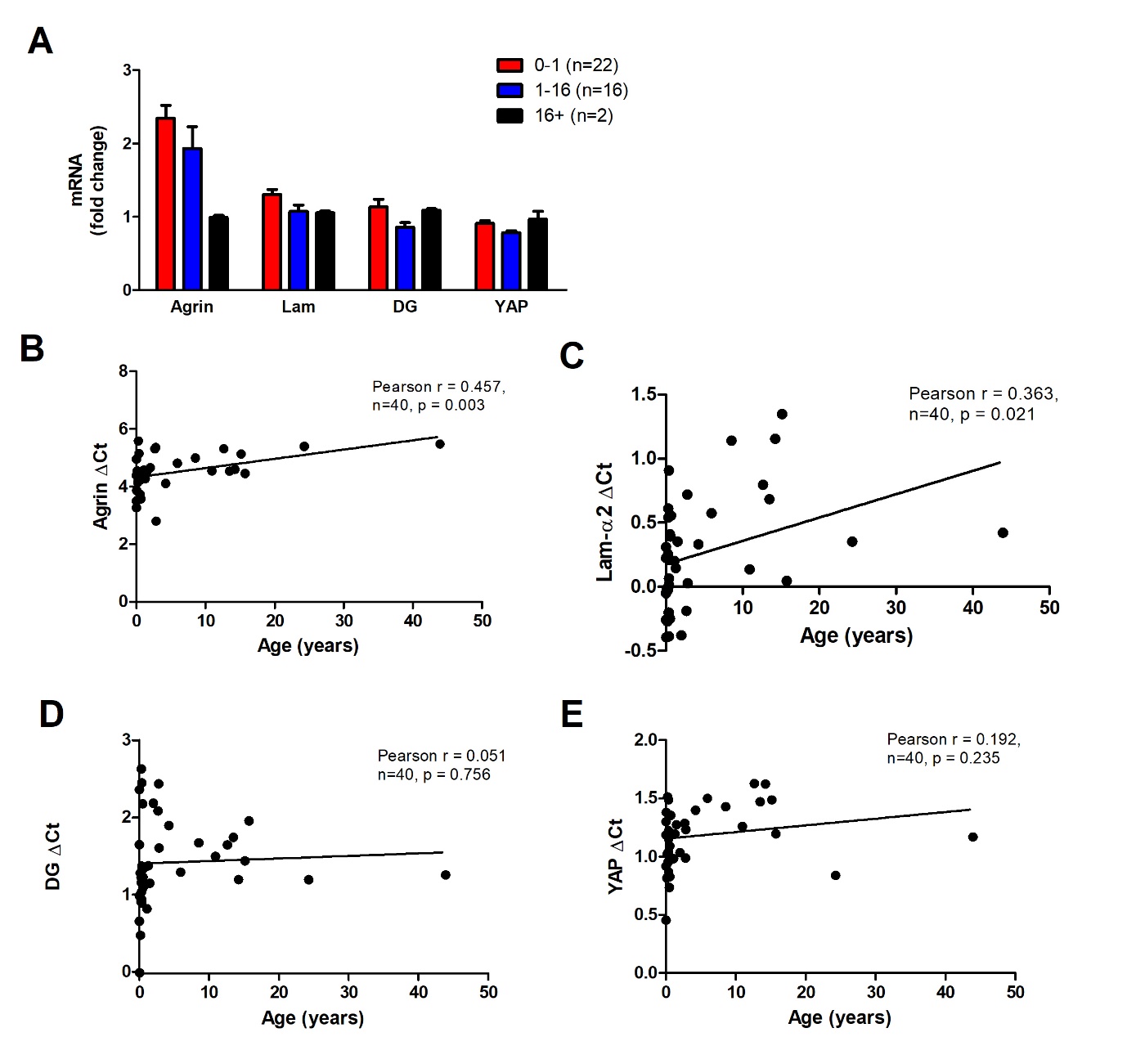
**

**Supplementary Figure S1. mRNA expression of agrin and associated proteins in human hearts.** Expression of agrin, laminin, DG and YAP (expressed as fold change relative to expression in the two adults) organized into three age groups (A). Correlation of the transcript levels of agrin (B), laminin (C), dystroglycan (DG; D) and YAP (E) with age in right ventricular biopsies from patients undergoing cardiac surgery (n=40), as normalized for 3 housekeeping genes, as described in the Methods section. Significant correlations were tested for using two-tailed Pearson correlation coefficient.

**Supplementary Figure S2. Quantification of laminin γ based on densitometry analysis.**

A decrease of protein levels with age is evident, with a reduction of 3.5 times by the age of 2 years. GAPDH was used as a normalizing control. See Figure 3C for reference.

Adult mouse


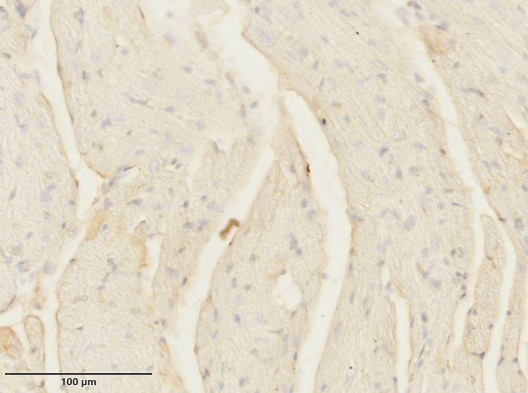


43 years old

8 years old

4.5 months old

6 days old

Control

(secondary Ab only)

Anti-laminin-α2 Ab


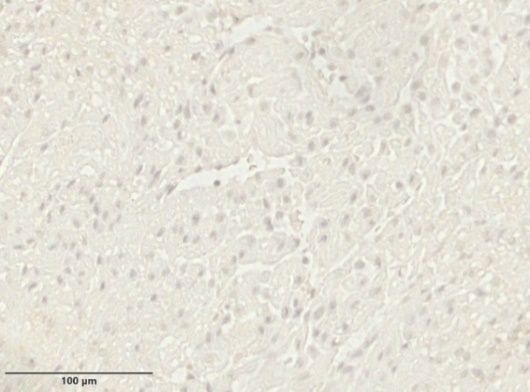

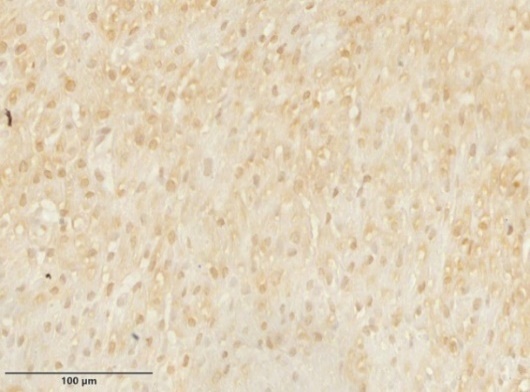

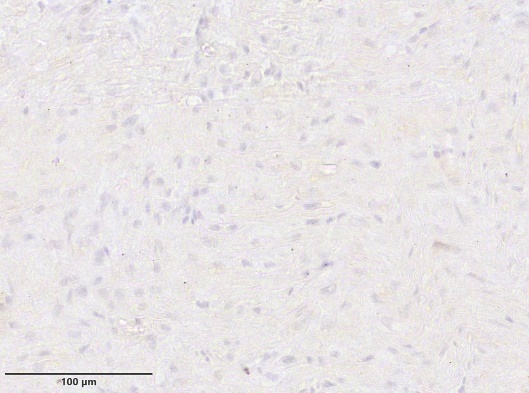

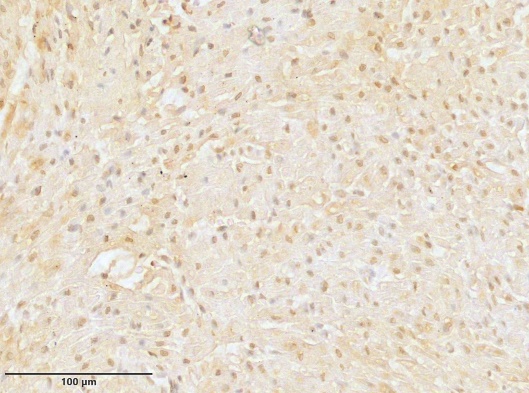

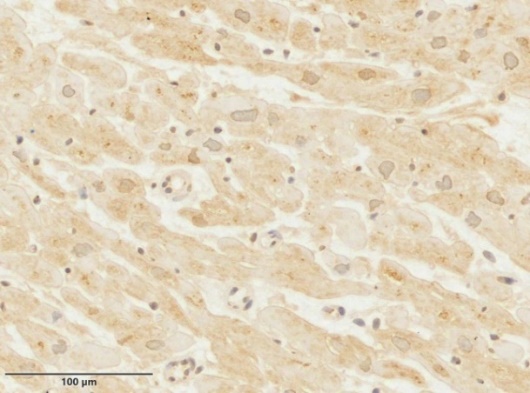

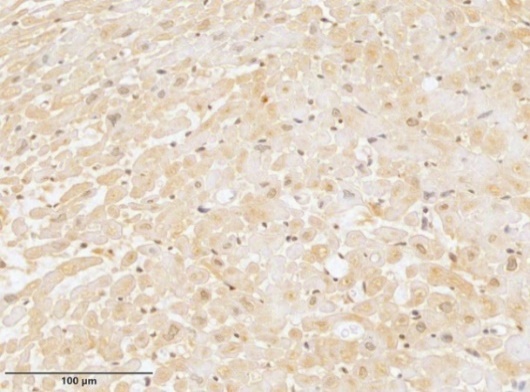

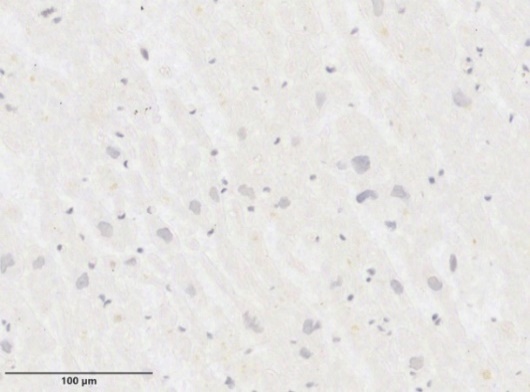

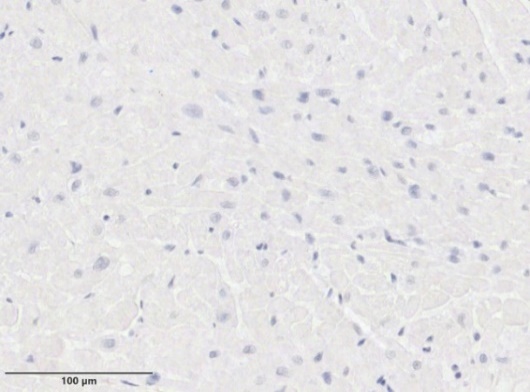

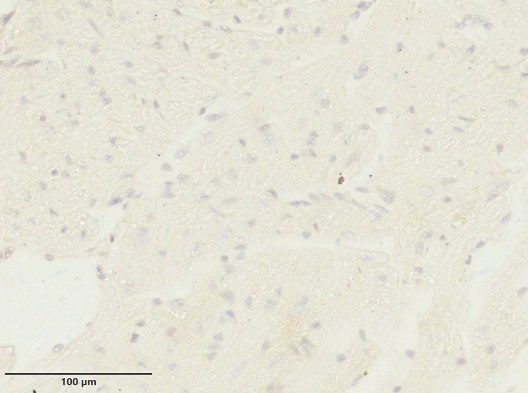


**Supplementary Figure S3. Immunohistochemical staining for laminin.** Immunohistochemical staining for laminin-α2 in the right ventricle of four patients aged between 6 days and 43 years and an adult mouse. Each scale bar represents 100µm. A negative control for each image is found in the right-hand column.

43 years old

8 years old


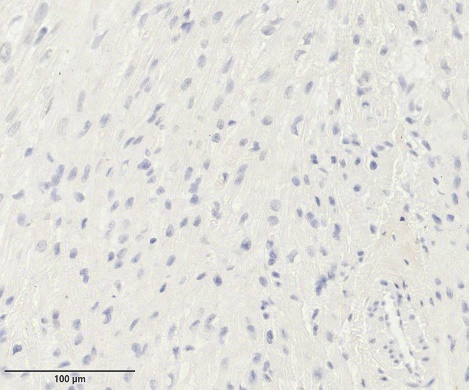

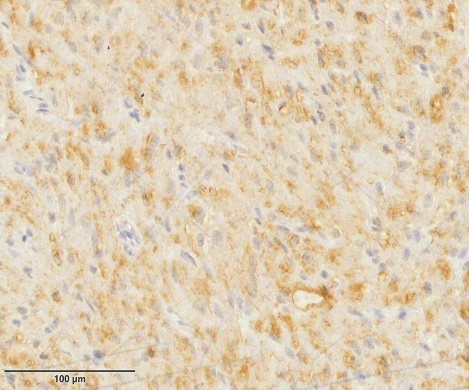


6 days old


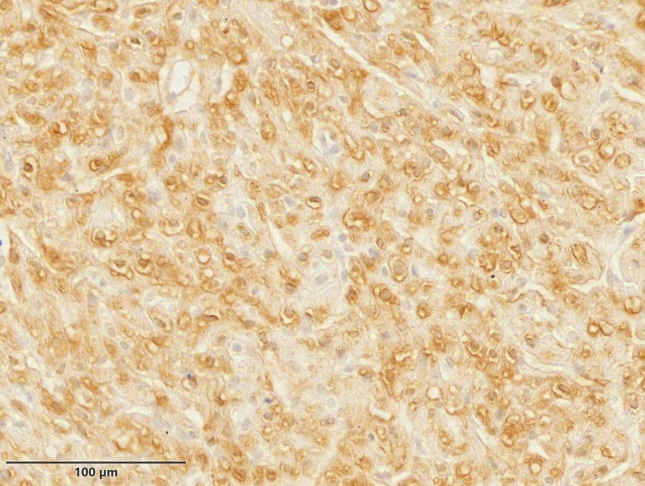

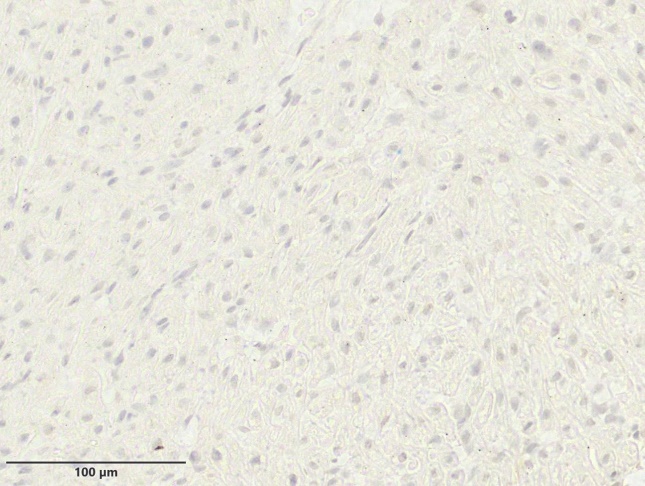

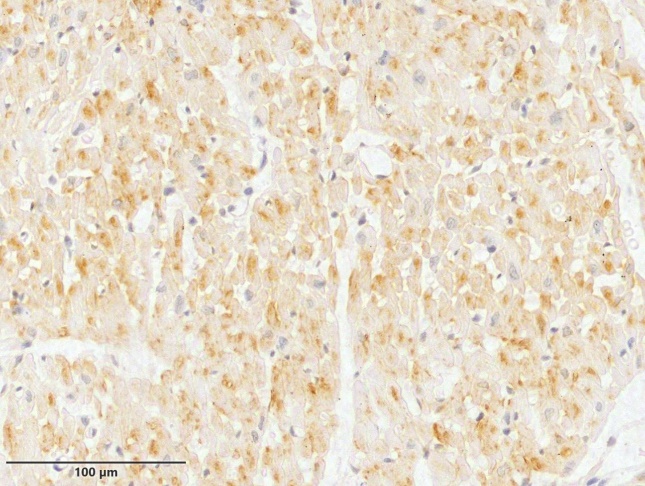

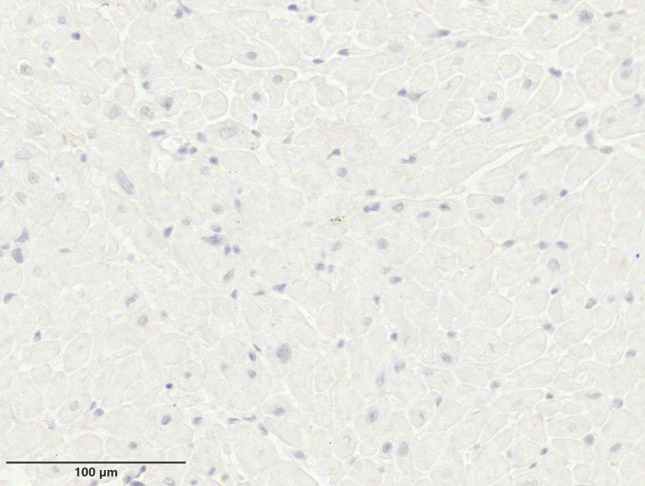

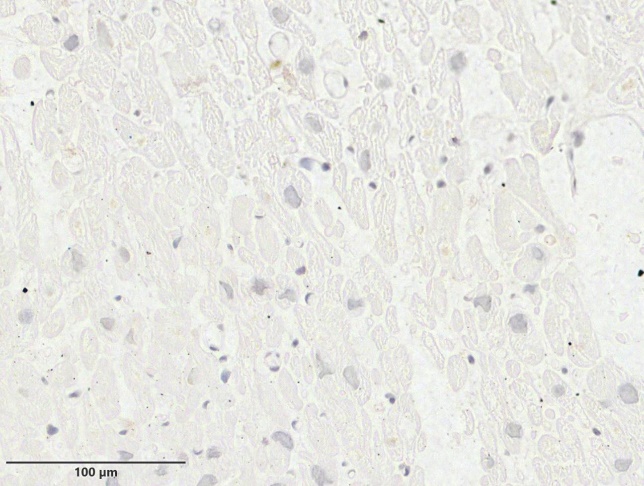

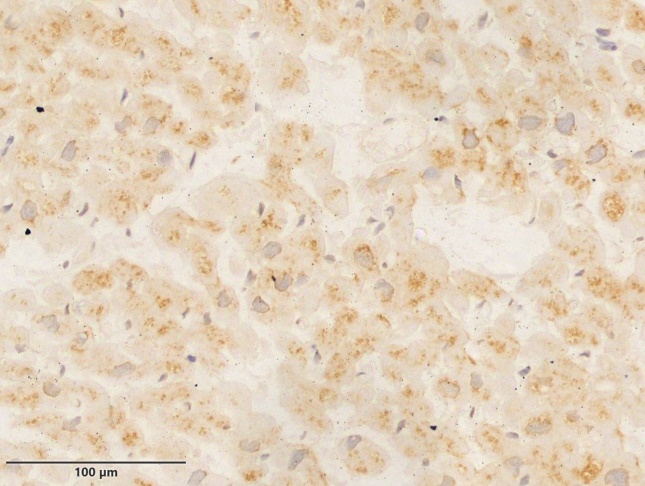


4.5 months old old

Anti-DG Ab

Control

(secondary Ab only)


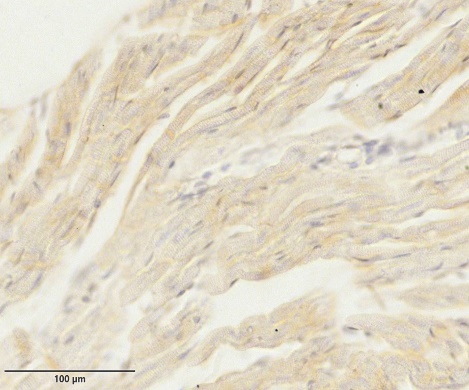

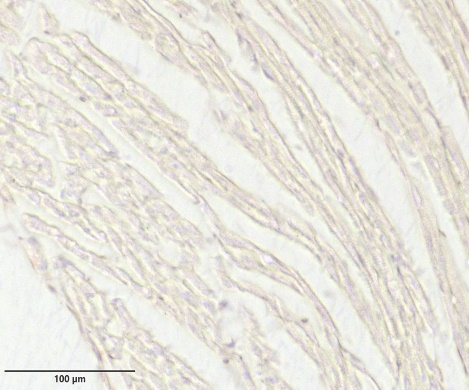


Adult mouse

**Supplementary Figure S4. Immunohistochemical staining for DG.** Immunohistochemical staining for DG in the right ventricle of four patients aged between 6 days and 43 years and an adult mouse. Each scale bar represents 100µm. A negative control for each image is found in the right-hand column
